# Diplopia as the Complication of Cataract Surgery

**DOI:** 10.1155/2016/2728712

**Published:** 2016-02-21

**Authors:** Maciej Gawęcki, Andrzej Grzybowski

**Affiliations:** ^1^Dobry Wzrok Ophthalmological Clinic in Gdansk, Kliniczna 1B/2, 80-402 Gdańsk, Poland; ^2^Department of Ophthalmology, University of Warmia and Mazury, Michała Oczapowskiego 2, 10-719 Olsztyn, Poland

## Abstract

The authors present systematic review of aetiology and treatment of diplopia related to cataract surgery. The problem is set in the modern perspective of changing cataract surgery. Actual incidence is discussed as well as various modalities of therapeutic options. The authors provide the guidance for the contemporary cataract surgeon, when to expect potential problem in ocular motility after cataract surgery.

## 1. Introduction

Cataract surgery is probably the most often performed surgical procedure in modern ophthalmology. Due to its commonness and improvement in surgical technique, the results are very spectacular, and so are patients' expectations, and any exception from this rule is categorised as surgical failure. Most of the complications that cataract surgeon faces in his practice are connected with the course of the surgery itself and usually there is a clear explanation why such a complication has occurred. Experienced surgeon is usually capable of assessing the difficulty of the procedure and therefore he usually informs the patient about possible complications and the amount of risk.

The situation is different with diplopia occurring after cataract surgery. It is not a frequent complication and usually appears as a major surprise and disappointment for both the patient and the surgeon. The purpose of our review is to assess the frequency of this complication and determine the group of patients at risk of developing diplopia after uncomplicated cataract surgery.

## 2. Methodology

We conducted a search through PubMed for prospective and retrospective studies on aetiology and incidence of postoperative diplopia in cataract surgery. We used the following key words: cataract surgery, diplopia, and disruption of fusion. For the final analysis we chose 50 articles dating from 1987 to 2014. We analysed the material according to incidence, aetiology, and treatment of diplopia after cataract surgery.

## 3. Incidence

Nowadays, there are not many reports in the literature that would cover the subject of postoperative diplopia in cataract patients. In 2006, Costa et al. reviewed 20453 records of patients that underwent cataract surgery in the period of 5.3 years ending in 2005 [1]. In this cohort of patients, only 19 (0.093%) reported double vision. The anaesthesia used during surgery was retrobulbar block ropivacaine diluted with hyaluronidase. Golnik et al. evaluated 118 consecutive patients operated on by one surgeon under retrobulbar anaesthesia. Change in ocular alignment in 7% of patients was reported, but only one patient out of 118 (0.85%) had symptomatic diplopia [2]. In 2009, Chung et al. analysed prospectively records of 160 consecutive patients who underwent cataract surgery in topical anaesthesia [3]. Among those patients only 7 (5%) complained of diplopia; however, they did not seek the medical assistance for its treatment. Yangüela et al. compared the incidences of diplopia after cataract surgery performed under topical or regional anaesthesia [4]. The series included 2122 patients operated on under regional anaesthesia and 1420 under topical anaesthesia. The incidence of postoperative diplopia was 0.99% (21 cases) in the regional group and 0.21% (3 cases) in the topical group. On average, the incidence was 0.68%. Pearce et al. extracted a group of 15 patients out of 4600 operated on for cataract, who had a vertical diplopia related purely to local anaesthesia (patients with preexisting strabological disorders were excluded), which makes the incidence of 0.3% [5]. As one can see from the above citations, the estimation of the incidence of diplopia after cataract surgery is quite difficult. Numbers given by different authors are not similar, so the only conclusion that can be made is that diplopia after cataract surgery seems to be really rare. The number of regional anaesthesia related cases of postsurgical diplopia is decreasing due to the use of topical medications so the subject of postoperative diplopia in cataract surgery is probably not in the focus of a contemporary surgeon.

Interesting data are obtained from strabological clinics treating diplopia.

Nayak et al. in 2008 reported a large number of patients (150) presenting with diplopia after cataract surgery, who were treated in orthoptic clinic during a period of 70 months between 1995 and 2000 [6]. All of the patients had a form of regional anaesthesia: peribulbar or retrobulbar. Some had also superior rectus stay suture applied during the surgery. Patients with postsurgical diplopia constituted 3% of all patients with diplopia treated in the clinic during that period. Similarly, Karagiannis et al. in 2007 reviewed records of 571 patients treated for diplopia over a period of 8 years [7]. Among them 39 patients (6.8%) had diplopia related to cataract surgery performed in peribulbar anaesthesia without use of hyaluronidase. Both studies go back a large span of time, when periocular anaesthesia was a rule. Presently for sure the percentage of patients treated for diplopia in orthoptic clinics after cataract surgery is much smaller, due to extensive use of solely topical anaesthesia during cataract surgery. Besides, in general, the number of patients with postoperative diplopia might be underestimated, due to their advanced age and lack of proper verbalization of this problem.

## 4. Aetiology

### 4.1. Surgical Technique and Local Anaesthesia

Most of the clinical reports concerning diplopia after cataract surgery date back 10 to 30 years. At that time, surgical technique was evolving from extracapsular cataract extraction to phacoemulsification. The first technique involved bridal suture for stabilization of the globe, which potentially could produce trauma to superior rectus muscle. The evolution in surgical technique influenced also the type of local anaesthesia used for cataract surgery. Retrobulbar anaesthesia used widely with extracapsular extraction involved penetration of the needle towards the muscle cone and naturally bared a number of possible complications, such as direct injury to the muscle or nerves, retrobulbar haematoma, muscle paresis, or toxic reaction to the anaesthetic itself. With peribulbar anaesthesia direct trauma to the muscle is much less likely to occur so motility disturbances after cataract surgery were in such cases attributed mainly to myotoxic effect of the anaesthetic. With the advent of purely topical anaesthesia in cataract surgery, both the myotoxic effect of the anaesthetic and direct injury to the muscles are practically excluded.

A few decades ago there were several reports published attributing postoperative diplopia after cataract surgery to the placement of bridal suture and subsequent injury to superior rectus or even superior oblique muscle [8–10]. From a modern perspective this case does not seem to be a problem anymore, as set sutures are presently not used for regular cataract surgery. However, history gives us knowledge about this possible complication in other ophthalmic surgeries involving the use of set sutures, such as glaucoma surgery or retinal surgery.

Majority of numerous reports concerning diplopia after cataract surgery published between 1990 and 2010 concentrate on the role of local anaesthesia in aetiology of that problem. At that time majority of cataracts were operated on in retrobulbar or peribulbar anaesthesia, which was administered by the injection of the anaesthetic to the retrobulbar or peribulbar region. In both cases the needle penetrated through the skin in the immediate neighbourhood of the extraocular muscles. The placement of the injection for the retrobulbar anaesthesia was inferolateral quadrant of the orbital aperture. For peribulbar region, usually 2 injections were performed, involving inferolateral and inferomedial regions of the orbit. In both techniques inferior rectus muscle was the most exposed muscle for the direct or indirect trauma. Some authors allow the possibility of direct injury to the muscle by the needle with anaesthetic resulting in muscle paresis or significant hematoma resulting in subsequent muscle ischemia followed by fibrosis. This explanation however seems to be less probable or less frequent than myotoxic action of the anaesthetic itself. What is interesting, in some opinion, is that myotoxic effect of injection of the anaesthetic to the muscle is more likely to occur with peribulbar anaesthesia. Esswein and von Noorden report 9 cases of vertical strabismus after cataract extraction, 7 of which were consistent with the form of peribulbar injection of the anaesthetic [11]. The usual composition of anaesthetic used in cataract surgery was 2% lidocaine and 0.5% bupivacaine with or without addition of hyaluronidase. Myotoxic action of the lignocaine and bupivacaine has been well documented in animal studies. Hyaluronidase is considered to have protective action against myotoxicity of the other agents [12, 13]. It helps spread the anaesthetic evenly through the tissues and prevents its focal high concentration, which bares the risk of toxic effect on the muscles. There are a few reports of rapid increase in the number of postcataract surgery diplopia cases when hyaluronidase became temporarily unavailable on the market in the late 90s [14, 15]. Taylor et al. report five cases of postoperative diplopia in which magnetic resonance scan was performed immediately after revealing the symptoms [16]. MRI showed swelling and increase in signal intensity in affected (paretic) muscle, which was interpreted as inflammatory oedema. There was no sign of intraorbital haemorrhage. This finding supports the toxic theory of local anaesthetic. Kim and Hwang report superior rectus contracture in patient with postsurgical diplopia visible on MRI scans in case of superior rectus overaction [17]. Animal studies showed rapid regeneration and no histological change of extraocular muscles after injection of just a simple saline solution [18].

The nature of anaesthesia related diplopia is predominantly vertical strabismus. Presentation is hypertropia or hypotropia, depending also on the time of examination. Patients with diplopia examined promptly after cataract surgery usually had hypertropia as a result of inferior rectus paresis. With time, hypertropia might switch to hypotropia due to inferior rectus fibrosis and contracture. This kind of scenario was observed by Capó and Guyton [19]. According to the authors, myotoxicity of the anaesthetic causes transient paresis of the muscle followed by contracture or fibrosis. Mild contracture results in overaction of the affected muscle and significant contracture results in restrictive strabismus.

Nayak et al. report approximately equal number of hypotropic and hypertropic patients; however, examination in most cases was performed six months after surgery [6]. Vlăduţiu et al. report four such cases, all hypertropic [20]. Similarly, Costa et al. [1] report most of the cases with hypertropic component of the ocular deviation. Other authors report predominantly restrictive vertical strabismus with inferior rectus involvement [21–23]. All these data confirm that inferior rectus is the most exposed for injury extraocular muscle during local anaesthesia. Injury to the superior rectus muscle has also been reported; however, these cases are much less frequent. There are several reports of oblique muscle injury due to anaesthesia in cataract surgery but these cases may be treated as anecdotal [24, 25].

An interesting question that emerged while analysing the problem was sidedness of the affected eyes. Deficient motility of the affected eye was noted more often on the left side. Most of the surgeons and anaesthetists are right handed and have more difficulty in performing peribulbar or retrobulbar anaesthesia in the left eye of the patient. The needle in such cases is directed more closely to the muscle cone [6, 26, 27]. The change in the technique of administering the local anaesthesia before cataract surgery resulted in significant reduction of postoperative diplopia cases [26]. Also, experience of the surgeon in performing this kind of anaesthesia evidently plays an important role in avoiding potential complications [27].

Most important aetiological causes of diplopia after cataract surgery in order of diminishing prevalence are as follows:

 Anaesthesia related.
 Preexisting strabological disorders, most often heterophoria and fourth nerve palsy.
 Optical and refractive complications.
 Sensory deprivation by dense cataract.

### 4.2. Optical or Refractive Reasons for Diplopia

Optical and refractive reasons for postoperative diplopia in cataract surgery are usually quite easy to determine. Lens implant decentration or subluxation can cause a monocular diplopia and require repositioning of the implant [28]. Another important question is anisometropia resulting in aniseikonia [29, 30]. This situation might happen in case of patients after cataract surgery in one eye, waiting for the operation of the other eye. Symptoms usually resolve after the second cataract operation. It may also happen in case of longstanding anisometropia corrected during cataract surgery by implantation of emmetropic IOL. Usually such patient requires a long process of postoperative adaptation and spectacle or contact lens correction. Another problem is anisometropia resulting from erroneous measurements of the IOL. This situation requires surgical correction: IOL exchange, piggy back IOL implantation, or refractive surgery.

### 4.3. Preexisting Disorders

Cataract can mask other disorders that can cause double vision by itself [31]. In that group we can include patients with retinal diseases in whom cataract makes the precise examination of the fundus impossible and reveal such disorders as epiretinal membranes, former intensive macular laser photocoagulation, or exudative macular degeneration. Silverberg et al. describe vertical diplopia in those conditions, corrected with success by Bangerter foil [32]. The other subgroup includes patients with preexisting conditions resulting in disturbed ocular motility, such as dysthyroid ophthalmopathy, motor nerve palsies, or myasthenia gravis. We can also experience a concurrent onset of such diseases. All those patients require systemic examination and treatment.

#### 4.3.1. Decompensation of Preexisting Heterophoria or Preexisting Strabismus and Fusion Disruption

Preexisting strabological disorders are the second reason after anaesthesia related trauma for postoperative diplopia in cataract surgery [6, 7, 33–35]. Cataract can disrupt fusion due to sensory deprivation and that process is more likely to occur with preexisting heterophoria. The most important question in such cases is whether fusion disruption is permanent and requires further treatment. Usually cataract patients are not carefully examined according to strabological disorders, so this group of disturbances might be easily overlooked during initial examination. Besides strabismus and phorias are diseases attributed mainly to childhood, so considering the average age of the cataract patient, medical history of these diseases may not be easy to collect. There are a few reports of strabismus formation due solely to dense cataract (patients without former history of strabismus) [36, 37]. In such patients authors report predominantly exodeviation and regain of binocularity after successful cataract surgery practically in all of them. It is very unusual for the cataract alone to disrupt fusion permanently. Patients with senile cataract may rarely experience disruption of fusion after the surgery: they cannot either suppress or fuse images [38, 39]. Symptoms are sometimes described as horror fusions. Usually patients spontaneously regain binocularity later. Sloper and Collins reported delayed visual evoked potentials in patients after dense cataract removal [40]. Measurements returned to normal approximately 3 months after surgery. Reports of permanent disruption of fusion in adults are rare [41]. Pratt-Johnson reports 2 such cases involving combination of a long standing traumatic cataract and unilateral uncorrected aphakia. The patients required surgery on extraocular muscles and optical correction and finally regained basic fusional ability. Reports of sensory deprivation by cataract seem to have permanent effect in children. Pratt-Johnson and Tillson in 1989 reported 24 cases of intractable diplopia after traumatic cataract removal in children [42]. The minimum time for cataract presence was 2.5 years. According to the author prognosis for regaining binocularity was poor.

Another question is the problem of postcataract diplopia in patients with preexisting strabismus, heterophoria, or amblyopia. It is well known that heterophoria can decompensate after optical correction, head trauma, or systemic disease. Dense cataract due to sensory deprivation disrupts fusion, so it may also be the cause for heterophoria decompensation. Besides, cataract surgery involves refractive error correction by the power of intraocular implant, so it bares the potential for the phoria deregulation. It is worth noting that decompensated phoria in long standing superior oblique nerve palsy frequently produces diplopia after cataract surgery [6, 7, 9]. In most cases of postcataract surgery diplopia due to strabological reasons the change is permanent and requires treatment, the same as in decompensated patients without cataract. Other causes of diplopia in that group include elimination of suppression in the amblyopic eye or change of fixation pattern, for example, in patients with abnormal retinal correspondence and monofixation syndrome. Nayak et al. report a large subgroup of patients with convergence insufficiency who were asymptomatic before the surgery [6]. Most of these patients required prismatic correction and orthoptic exercises.

## 5. Treatment

Treatment of diplopia after cataract surgery involves conservative approach and surgical procedures. The choice of treatment depends on aetiology of diplopia, so the key factor is to determine it early. Sometimes magnetic resonance imaging of the extraocular muscles helps in determining the cause of postoperative deviation [43]. Most of the ophthalmologists wait for a few months after cataract surgery for the symptoms to recede. A number of patients regain fusional ability within a few months after cataract extraction, so it is sensible to wait for a while before taking into account such serious measures as surgical treatment. Statistically speaking majority of treated patients benefit from conservative treatment (depending on the author, 60–80%) [6, 7, 33, 44]. It can be applied early after the onset of symptoms and includes first of all prismatic correction, usually Fresnel prisms. Prismatic correction can be used as a sole solution or temporary measure before the surgical treatment [45]. It works particularly well in patients with small comitant deviations. Sometimes orthoptic exercises help, for example, in convergence insufficiency or temporary disruption of fusion. The other less invasive procedures are injections with botulin toxin into hyperactive muscle [22]. Surgical treatment of postcataract diplopia is applied as the second choice, when the deviation is stable. It is the only solution in patients with muscle paresis and/or muscle contracture. The surgical procedures are usually performed on vertical muscles: inferior rectus or superior rectus recessions [21, 46]. Occasionally inferior rectus plication may be performed [47]. In cases with the presence of horizontal component of deviation, horizontal muscles are also operated on [1]. Adjustable sutures technique is particularly recommended [48, 49].

## 6. Conclusions

Diplopia after cataract surgery does not seem to be a major problem for a cataract surgeon nowadays. Its incidence was always probably much less than 1% and has definitely diminished with the progress in modern cataract surgery techniques. Due to the use of topical medication only for cataract surgery, the question of myotoxic action of the anaesthetic is now a historical problem. Besides, cataracts are now operated on much earlier than it was 20 years ago and dense cataract that could disrupt fusion or cause permanent sensory deprivation is rarely seen in the western countries. However, diplopia after cataract surgery might occur in young patients with posttraumatic cataracts. This review shows that besides anaesthesia related diplopia the most frequent diplopia after cataract surgery affects patients with this kind of disorders in the past. It is sensible then to include history of such disorders in the preoperative questionnaire. It seems also reasonable to cooperate with orthoptic clinics in case of such complication. Results of treatment of diplopia after cataract surgery are generally good and there are very few cases when diplopia becomes a nuisance for a patient. Conservative treatment works well in most cases, but in surgical procedures adjustable sutures provide the most predictable effects.
